# Phosphate depletion modulates auxin transport in *Triticum aestivum* leading to altered root branching

**DOI:** 10.1093/jxb/eru284

**Published:** 2014-08-02

**Authors:** Peter J. Talboys, John R. Healey, Paul J. A. Withers, Davey L. Jones

**Affiliations:** School of Environment, Natural Resources and Geography, Deiniol Road, Bangor, Gwynedd LL57 2UW, UK

**Keywords:** AUX/IAA, auxin, lateral root, phosphate, PIN, polar auxin transport, wheat.

## Abstract

Understanding the mechanisms by which nutritional signals impact upon root system architecture is a key facet in the drive for greater nutrient application efficiency in agricultural systems. Cereal plants reduce their rate of lateral root emergence under inorganic phosphate (Pi) shortage; this study uses molecular and pharmacological techniques to dissect this Pi response in *Triticum aestivum*. Plants were grown in coarse sand washed in high- or low-Pi nutrient solution before being assessed for their root branching density and expression of *AUX/IAA* and *PIN* genes. Seedlings were also grown on media containing [^14^C]indole acetic acid to measure basipetal auxin transport. Seedlings grown in low-Pi environments displayed less capacity to transport auxin basipetally from the seminal root apex, a reduction in root expression of *PIN* auxin transporter genes, and perturbed expression of a range of *AUX/IAA* auxin response genes. Given the known importance of basipetally transported auxin in stimulating lateral root initiation, it is proposed here that, in *T. aestivum*, Pi availability directly influences lateral root production through modulation of *PIN* expression. Understanding such processes is important in the drive for greater efficiency in crop use of Pi fertilizers in agricultural settings.

## Summary

When grown in a low-phosphate environment *Triticum aestivum* showed reduced basipetal auxin transport and altered root PIN and AUX/IAA expression profiles, with a concurrent reduction in root branching density.

## Introduction

The plasticity of root system architecture in response to environmental cues is a crucial component of a plant’s nutrient foraging capacity. The production of lateral root branches is genetically controlled and may increase root surface area in nutrient-rich soil (Drew, 1975; Linkohr *et al.*, 2002), or enable the exploration of a greater soil volume by lateral growth through the topsoil in nutrient-poor soil (Linkohr *et al.*, 2002; Zhu and Lynch, 2004). An example of this process is the acquisition of inorganic phosphate (Pi), in which the production of lateral roots is crucial for Pi accumulation in some plants (Lynch, 2011). The factors controlling root branching to form new lateral roots are therefore of great interest, and are the focus of this study.

Phosphate is an essential plant nutrient required for photosynthesis and a key building block in biological molecules such as nucleic acids and phospholipids. The concentrations of Pi in soil solution are, however, typically very low, due to Pi’s propensity to bind strongly to soil surfaces or form insoluble complexes with cations (Norman and Hemwall, 1957). This means that Pi is often a limiting factor in plant growth and development. This has resulted in a large number of developmental traits amongst plant species that can enhance Pi uptake. Physiologically these include the modulation of root elongation (Sánchez-Calderón *et al.*, 2005), branching (Linkohr *et al.*, 2002; López-Bucio *et al.*, 2002), and root hair density (Ma *et al.*, 2001). The root system may also act to enhance Pi uptake by exuding protons (Hinsinger, 2001), organic acid anions (Ryan *et al.*, 2001), and phosphatases (Tadano and Sakai, 1991) into the rhizosphere, or by the formation of symbioses with arbuscular mycorrhizas or ectomycorrhizas (Péret *et al.*, 2011; Smith *et al.*, 2011). Understanding the mechanisms controlling these traits is therefore of great importance in the pursuit of improved crop Pi uptake. The wheat crop is a major source of cereal for the world’s expanding population, and this work investigates the response of the root system of the crop plant spring wheat (*Triticum aestivum*) to Pi deficiency.

Work on the model plant *Arabidopsis thaliana* has been very successful in determining the sequence of molecular and cellular processes behind lateral root production. Primed pericycle founder cells, formed in the basal root meristem and located opposite xylem poles (Dolan *et al.*, 1993), undergo several rounds of ordered asymmetric cell division to form dome-shaped lateral root primordia (LRP) which then emerge from the parent root (Dubrovsky *et al*., 2001, 2008, 2011; De Smet *et al.*, 2006; De Rybel *et al.*, 2010; Moreno-Risueno *et al.*, 2010). The spatial distribution of lateral root production is a tightly controlled process, in which the phytohormone auxin plays a key role.

At the root apex, auxin distribution is tightly controlled by the differential expression and subcellular localization of the AUXIN RESISTANT (AUX) and PIN-FORMED (PIN) auxin carrier proteins which mediate influx and efflux, respectively, in a process known as polar auxin transport (PAT) (Palme and Gälweiler, 1999). The protein AtPIN1 unloads leaf-derived auxin from the vascular tissue into the root apical meristem (RAM) (Gälweiler *et al.*, 1998), where AtPIN3, AtPIN4, and AtPIN7 proceed to create auxin maxima in both the quiescent centre cells at the heart of the RAM and in the collumella root cap distal to it (Friml *et al*., 2002*a*, b, 2003). The expression of *AtPIN2* and *AtAUX1* in lateral root cap cells and *AtPIN2* epidermal cells then drives a basal flow of auxin on the root periphery (Müller *et al.*, 1998). This basipetal transport of auxin in the lateral root cap and epidermis is crucial for auxin accumulation in the basal portion of the RAM. This is the driver of both gravitrophism (Abas *et al.*, 2006) and, importantly for this study, lateral root formation (Casimiro *et al.*, 2001; De Smet *et al.*, 2006). In the basal RAM, basipetally transported auxin accumulates in groups of pericycle cells, which have been specified by oscillating gene expression, to and from primed pericycle founder cells (Dubrovsky *et al*., 2001, 2008, 2011; De Smet *et al.*, 2006; De Rybel *et al.*, 2010; Moreno-Risueno *et al.*, 2010). These founder cells retain many cytological features characteristic of meristematic cells (dense cytoplasm, large nuclei, and small vacuoles) and maintain a level of multipotency whilst the remainder of the root tissue differentiates around them (Dubrovsky *et al.*, 2008; Parizot *et al.*, 2008). Genes related to the cell cycle are subsequently triggered in these founder cells, and so the cell division events which drive the formation of LRPs are also induced by auxin (Himanen, 2002; Himanen *et al.*, 2004; Dubrovsky *et al.*, 2008).

The majority of auxin signal transduction is known to require three major protein components: AUX/IAA transcriptional repressors (Abel and Theologis, 1996), AUXIN RESPONSE FACTOR (ARF) transcriptional activators (Guilfoyle and Hagen, 2007), and the Skp1-cullin-F box protein E3 ubiquitin ligase (SCF) and its F box component TRANSPORT INHIBITOR RESPONSE 1 (TIR1) (Dharmasiri *et al.*, 2005; Kepinski and Leyser, 2005). Briefly, in the absence of auxin, AUX/IAAs bind to ARFs and prevent them from activating transcription of auxin-responsive genes. Auxin acts as a molecular glue, stabilizing the direct interaction between TIR1 and the AUX/IAA (Dharmasiri *et al.*, 2005; Kepinski and Leyser, 2005). This enables the SCF complex to ubiquitinate the AUX/IAA, targeting it for degradation (Gray *et al.*, 2001), and thus allows the ARF to activate transcription (Dharmasiri *et al.*, 2005; Kepinski and Leyser, 2005). Auxin signalling also involves a reverse feedback loop, whereby *AUX/IAA* genes are among those whose expression is activated by ARFs: thus auxin signal transduction is a tightly restricted process, and *AUX/IAA* expression correlates well with increased auxin concentrations (Vanneste and Friml, 2009). A number of *AUX/IAA* genes have been linked to lateral root initiation and development in *Arabidopsis*: *AtIAA28* regulates founder cell specification (De Rybel *et al.*, 2010), *AtIAA14* regulates the asymmetric divisions that form the first committed steps in lateral root production (De Smet *et al.*, 2010), and *AtIAA12* and *AtIAA13* also participate in lateral root development subsequent to *AtIAA14* (Goh *et al.*, 2012).

Greater production of lateral roots has been shown to increase Pi acquisition efficiency substantially (Zhu and Lynch, 2004), and there is a variation amongst plant species in how lateral root production is used to maximize Pi uptake in low-Pi environments (Niu *et al.*, 2013). *Arabidopsis*, *Brassica nigra*, and *Hordeum vulgare* root systems have been reported to respond to homogeneously low-Pi environments by promotion of lateral growth at the expense of vertical growth (Linkohr *et al.*, 2002; Huang *et al.*, 2008). Here the primary RAM terminally differentiates (Sánchez-Calderón *et al.*, 2005), resulting in the cessation of root growth, and an increase in the frequency of lateral root initiation and lateral root elongation (Linkohr *et al.*, 2002; López-Bucio *et al.*, 2002). However, conflicting reports demonstrate that, subsequent to longer term exposure to low Pi conditions, *Arabidopsis*, *H. vulgare*, and *Phaseolus vulgaris* root systems show reductions in lateral root branching density (Drew, 1975; Borch *et al.*, 1999; Nacry *et al.*, 2005). In *Arabidopsis*, this temporal contrast is proposed to be caused by low Pi conditions stimulating the emergence of existing LRPs, yet reducing the overall number of primordia generated (Nacry *et al.*, 2005). A contrast can, however, be drawn between *Arabidopsis* and *H. vulgare* in their reactions to localized areas of high soil Pi: *Arabidopsis* shows no branching response to these Pi patches (Linkohr *et al.*, 2002), whereas *H. vulgare* responds by significantly increasing branching frequency (Drew, 1975). This difference between the branching responses of these dicot and monocot species to Pi supply highlight the potential hazards of extrapolating developmental responses to nutrient availability between species that differ in their morphology, physiology, and phylogenetic history. Monocot cereals have a fine fibrous root system composed of multiple seminal and crown roots, rather than a tap root. This results in greater exploration of the topsoil than in the tap root system of the model plant *Arabidopsis*; therefore, the cereal root system as a whole encounters a more diverse range of nutritional environments (Hodge, 2009). This is especially important for Pi given its lack of mobility in soil solution.

This study uses spring wheat (*Triticum aestivum*) as a model to investigate how cereal root systems respond to variable Pi availability at a molecular and physiological level. This crop was selected because of its agronomic importance, the inaccuracy of extrapolating responses between species, and the lack of studies focused on the molecular mechanisms behind such processes in cereals. Despite its crucial role in global food production, the complex nature of the *T. aestivum* genome means that, until recently, very few studies have focused on the molecular basis of its developmental plasticity.

## Materials and methods

### Growing conditions

*Triticum aestivum* L. (cv. Paragon) seeds were surface-sterilized for 5min in a solution containing 10% Na hypochlorite and 0.01% Tween-20 (w/v). These seeds were then germinated on autoclaved tissue paper, moistened with sterile de-ionized water, for 3 d. The resulting seedlings were then planted in 50ml polypropylene tubes filled with autoclaved, washed quartz sand, and the whole system was watered to field capacity with an adapted Hoagland’s nutrient solution (Hoagland and Arnon, 1950). Water losses due to plant uptake and evaporation reduced the water content of the sand by around one-third over 24h; therefore, it is unlikely that flooding of the root system could be a confounding factor. Sand culture was used to minimize solid phase phosphorus interactions and the release of native phosphorus from soil organic matter, and to facilitate recovery of intact root systems. The Hoagland’s solution contained: 5mM KNO_3_; 5mM Ca(NO_3_)_2_; 2mM MgSO_4_; 765nM ZnSO_4_; 320nM CuSO_4_; 46.3 μM H_3_BO_3_; 497 μM Na_2_MoO_4_; 9.14 μM MnCl_2_; 1mM NH_4_NO_3_; 38.7 μM Fe.EDTA; and either 500 μM KH_2_PO_4_ (high-Pi) or 5 μM KH_2_PO_4_ and 495 μM KCl (low-Pi) (all Sigma Aldrich, Poole, UK). The solution pH was adjusted to 6.0 and autoclaved before use. Solutions containing the synthetic auxin 2,4-dichlorophenoxyacetic acid (2,4-D) and the PAT inhibitor 2,3,5-triiodobenzoic acid (TIBA) (both Sigma Aldrich) were 0.2 μm filter sterilized and then applied to the nutrient solution after autoclaving. 2,4-D was used rather than the endogenously produced auxin indole acetic acid (IAA) due to its increased stability over the time span of the experiment. The tubes were kept in a climate-controlled cabinet at 20 °C, 70% humidity, 16h/8h day/night cycle, and light intensity (PAR) of 500 μmol m^–2^ s^–1^, with a randomized layout. The tubes were re-watered daily to field capacity with the relevant nutrient solution. Seven days after planting (10 d after germination), the tubes were emptied and the roots were washed to remove any sand prior to subsequent analysis. Depending upon the experiment, the root systems were then either assayed for their lateral root branching density and longest lateral root length, or 3cm lengths of five seminal roots and five lateral roots per sample were frozen in liquid N_2_ for future quantitative PCR (qPCR) analysis. Root branching frequency was assayed on washed roots by first measuring the distance between the oldest and the newest, emerged, lateral root, and then counting the frequency of lateral root branches from the seminal root axis. Only the longest seminal root of the 3–5 present per plant was used. The longest lateral root length was determined using a ruler. The initial seminal root growth rate was measured under the same conditions as described above: each seedling’s seminal roots were, however, measured prior to planting, and then seedlings were harvested 24h later and the seminal roots re-measured to find the daily growth rate. All statistical significance testing was performed using Student’s *t*-test on MS Excel.

### Quantitative reverse transcription–PCR (RT–PCR)

RNA was extracted from the liquid N_2_-frozen harvested roots. Briefly, the first 1cm of root tip was excised using a scalpel and 10 root tips were pooled per extract. Each root tip was excised from a separate plant, and each pool of 10 was treated as one biological replicate. These were flash-frozen in liquid N_2_ and then the RNA was extracted, using a GeneMATRIX RNA/miRNA purification kit (Roboklon, Berlin, Germany) as per the manufacturer’s instructions. The dART RT kit (Roboklon) was then used to construct cDNA from this RNA extract using oligo d(T) primers. The target genes for qPCR analysis were obtained by performing a tblastn search of both the NCBI and the TIGR online databases using the protein sequences of AtPIN2 and AtIAA2. These sequences are referred to herein as: *IAA2* (GenBank: CK213604), *IAA3* (GenBank: CK170519), *IAA4* (GenBank: CK163783), *IAA5* (GenBank: BI751049), *IAA6* (GenBank: AK332471), *IAA7* (GenBank: AK331670), *IAA8* (GenBank: AK330790), *PIN3* (GenBank: CK208792), and *PIN4* (GenBank: CK208849). These candidates, alongside the previously uploaded sequences of *IAA1* (GenBank: AJ575098), *PIN1* (GenBank: AY496058), and *PIN2* (BK005137), were assessed to ensure that they all contained characteristic *PIN* or *AUX/IAA* domains (Supplementary Figs S1, S2 available at *JXB* online) by first using the ExPASy translate tool (web.expasy.org) to determine their amino acid sequences, aligning them using MUSCLE (www.ebi.ac.uk), then using TMPred (Hofmann and Stoffel, 1993) to predict transmembrane helices. Primers were designed using the NCBI primer blast tool (Supplementary Table S1), produced by Eurofins (Eurofins MWG-Operon, Ebersberg, Germany), and tested for specificity by performing standard PCR on cDNA extracts and performing electrophoresis on agarose gels. Quantitative RT–PCR was performed using a thermocycler (Applied Biosystems, Life Technologies Ltd, Paisley, UK) and SYBR Green qPCR mix (Roboklon), and normalized to actin (GenBank: AB181991) and tubulin (GenBank: U76558) controls performed using primer pairs published by Teng *et al.* (2013) and Zhang *et al.* (2012). Normalization was performed by dividing the relative expression values for each sample by the square root of the product of that sample’s actin and tubulin relative expression values. A further set of quantitative RT–PCR assays were performed on cDNA extracted from the root tissue of plants submerged in high-Pi media ±1 μM 2,4-D for 1h, having been grown to 10 d after germination as above, to ensure that their expression showed the auxin-responsive increase in transcription expected of *AUX/IAA* genes (Supplementary Fig. S3). All statistical significance testing was performed using Student’s *t*-test on MS Excel.

### [^14^C]IAA transport assays

PAT assays were conducted by a method adapted from Mishra *et al.* (2009). Seeds were surface-sterilized as previously described, and then germinated in Petri dishes containing the high-Pi Hoagland’s solution (described above) solidified with 10% Agar agar (Sigma Aldrich). Environmental conditions were as described earlier. Split Petri dishes were then created, with one half containing high or low-Pi Hoagland–agar medium previously described, and the other half containing either high- or low-Pi Hoagland–agar medium supplemented with 50nM of the endogenous auxin IAA labelled with ^14^C (American Radiolabelled Chemicals Inc., St. Louis, MO, USA). Two days after germination, seedlings were transferred to these split plates so that the first 1mm of the longest seminal root’s tip was in contact with the agar containing [^14^ C]IAA, with a 1mm gap between the [^14^C]IAA-containing agar and the non-radioactive agar with which the remainder of the root, and root system was in contact. The [^14^C]IAA-containing agar did not contact the non-radioactive agar. These seedlings were left for 1h at 20 °C. The roots were then dissected so that 2×2mm sections were taken from immediately behind the 1mm that was in contact with the agar containing [^14^C]IAA. These sections were oven dried at 105 °C for 24h, and their ^14^C content was then determined with an OX-400 Biological Sample Oxidizer (RJ Harvey Instrument Corp., Hillsdale, NJ, USA) with the ^14^CO_2_ evolved collected in Oxosol scintillation fluid (National Diagnostics, Hessle, UK). Four root sections were pooled per replicate to ensure a sufficient ^14^C signal, with three such replicates performed per treatment. ^14^C was then quantified using a Wallac 1404 scintillation counter (Wallac EG&G, Milton Keynes, UK). The ratio of [^14^ C]IAA content between the 2mm section closest to the root tip and the 2mm section immediately basal to it was used as an approximate estimate of relative auxin flow. These values were scaled to be proportionate to the high tip Pi, high basal Pi environments value. Replicates exposed only to agar containing no added [^14^C]IAA displayed no measurable ^14^C signal. Statistical significance testing was performed using Student’s *t*-test on MS Excel, and two-way analysis of variance (ANOVA) in SPSS.

## Results

### *Triticum aestivum* branching frequency reduces in low-Pi environments yet remains auxin sensitive

**Fig. 1.**
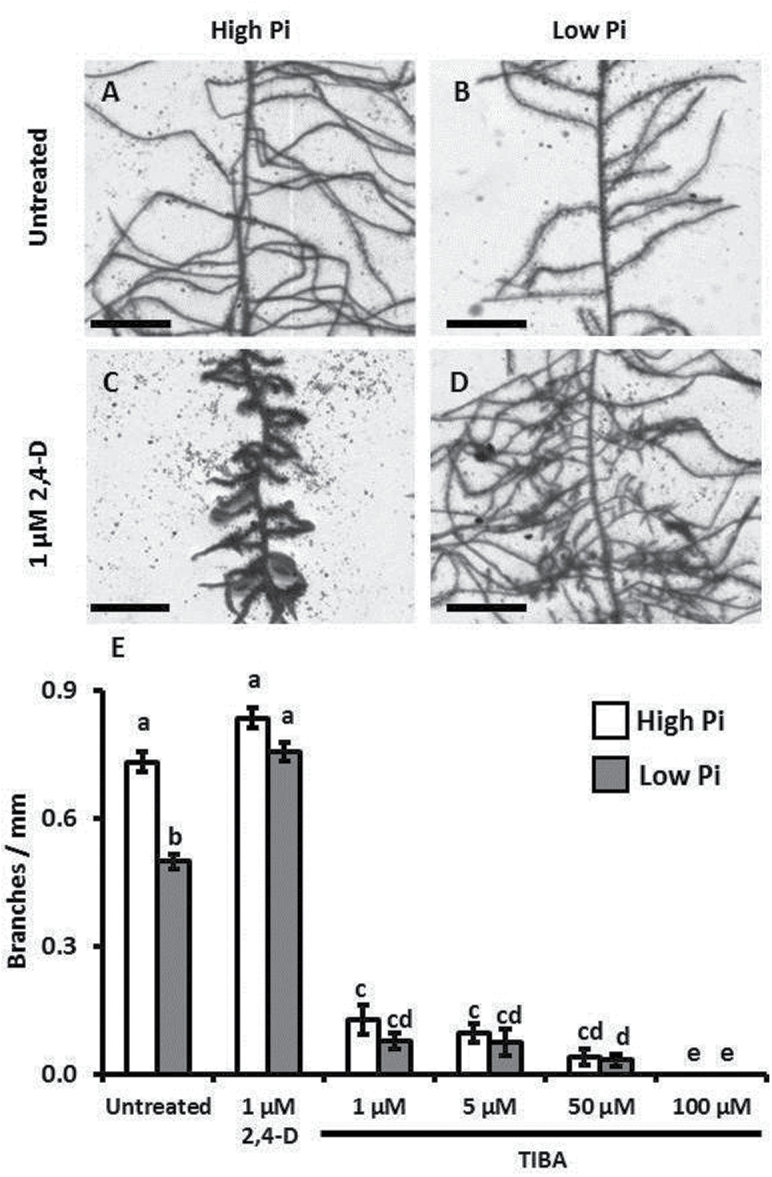
*Triticum aestivum* lateral root production in low-Pi is increased by auxin, and Pi-induced root branching requires polar auxin transport. (A–D) Images of *T. aestivum* roots grown for 7 d in sand culture (10 d after germination) watered with a nutrient solution containing (A, C) 500 μM or (B, D) 5 μM Pi, supplemented with (C, D) 1 μM 2,4-D. Scale bars are 1cm. (E) Frequencies of lateral root branches per millimetre of seminal root axis. Error bars are the standard errors of the mean (SEM), *n*=47–52, a, b, c, d, and e denote values significantly different from each other using Student’s *t*-test (*P*<0.05).

**Fig. 2.**
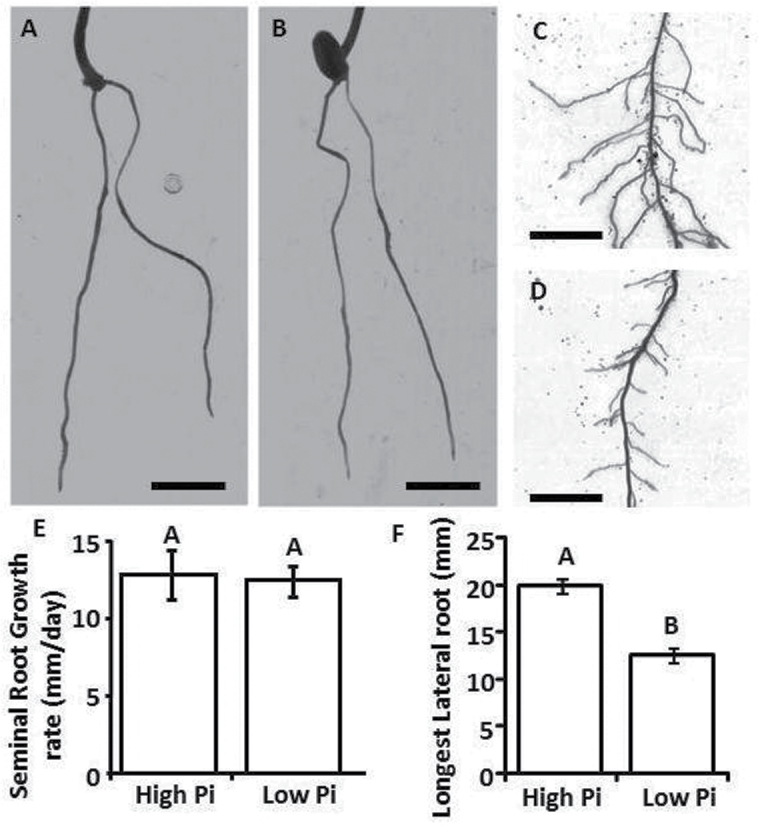
Lateral roots but not seminal roots show reduced length in low-Pi conditions. (A, B) Roots of *Triticum aestivum* seedlings grown for 3 d in sand culture watered with a nutrient solution containing (A) 500 μM or (B) 5 μM Pi. (C, D) *T. aestivum* roots grown for 10 d in washed sand watered with a nutrient solution containing (C) 500 μM or (D) 5 μM Pi. (E) Seminal root growth rates of seedlings from A and B. (F) Longest lateral root lengths found after 10 d growth in sand in a nutrient solution containing 500 μM Pi (high-Pi) or 5 μM Pi (low-Pi). Error bars are the SEM (*n*=15 for seminal root growth rates, *n*=45 root systems for longest lateral root assay). Letters indicate significant differences between high and low-Pi values using Student’s *t*-test (*P*<0.05).

As previously demonstrated for *H. vulgare* root systems (Drew, 1975), *T. aestivum* seminal roots produced a lower frequency of lateral roots in low-Pi environments than when exposed to high concentrations of Pi (Fig. 1A, B). Alongside this observation, initial seminal root growth rates were unaffected by environmental Pi supply (Fig. 2A, B, E), whereas low-Pi conditions resulted in a significant limitation in maximum lateral root length (Fig. 2C, D, F).

Seedlings grown in low-Pi media supplemented with 1 μM 2,4-D demonstrated a significant recovery in root branching frequency, demonstrating that they retained the capacity to respond to exogenous auxin (Fig. 1C, D). Interestingly, seedlings grown under high-Pi and at this dosage of 2,4-D demonstrated a drastic reduction in lateral root elongation, a characteristic of auxin application, whereas the low-Pi+2,4-D seedlings showed levels of lateral root elongation more similar to the no auxin controls (Fig. 1A, B). The inclusion of 100 μM TIBA (an auxin transport inhibitor) in the growth media showed that inhibition of auxin transport could severely reduce lateral root outgrowth (Fig. 1E), a similar response to that found in other plant species (Karabaghli-Degron *et al.*, 1998). The 1, 5, and 50 μM TIBA treatments allowed lateral root outgrowth, whilst also showing no significant effect of environmental Pi concentration on lateral root density. Therefore these data suggest that unimpeded PAT is required for Pi-mediated modulation of lateral root density.

### Expression of putative *AUX/IAA* genes is perturbed in response to environmental Pi

**Fig. 3.**
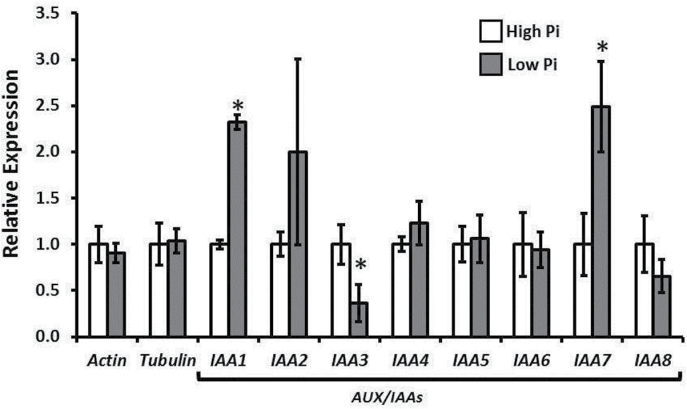
Expression of *AUX/IAA* genes is altered in response to environmental Pi. Relative expression levels of *AUX/IAA* gene candidates in *Triticum aestivum* seedlings grown in either low-Pi (5 μM) or high-Pi (500 μM). Each sample was normalized for actin and tubulin expression as detailed in the Materials and methods, and expression of *AUX/IAA* genes is plotted as a value relative to each gene’s high-Pi value. Values are the averages of three biological replicates (10 pooled root tips per replicate), with each of these being a pooled average of three experimental replicates. Error bars are the SEM (*n*=3). Asterisks indicate where low-Pi values are significantly different from the high-Pi values within each gene using Student’s *t*-test (*P*<0.05).

Bioinformatic analyses identified eight matches with predicted protein sequences with a highly similarity to the AtIAA2 probe used. These sequences all demonstrated domains III and IV characteristic of AUX/IAA sequences, and either also possessed domains I and II or were incomplete sequences (Supplementary Fig. S2 at *JXB* online). Expression levels of three of the seven identified potential *AUX/IAA* genes were significantly altered by the Pi status of the growth media (Fig. 3). The expression of *IAA1*, *IAA4*, and *IAA7* was significantly up-regulated under low-Pi conditions (Fig. 3), which contrasts with the reduced sensitivity of root elongation to exogenous auxin (Fig. 1C, D). However, the expression of *IAA3* was significantly reduced under low Pi.

### Basipetal auxin flow is reduced under phosphorus starvation, as is the expression of putative PINs

**Fig. 4.**
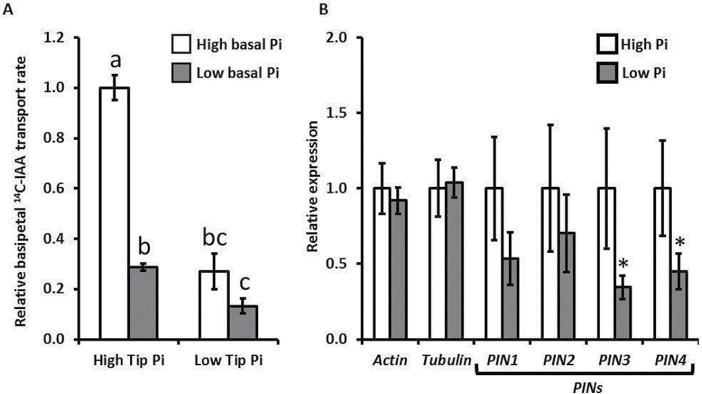
Polar auxin transport and expression of candidate genes for PIN-FORMED (PIN) auxin carrier proteins are down-regulated in low Pi. (A) Relative IAA transport rates measured by using a [^14^C]IAA label for *Triticum aestivum* seedlings with root tips in contact with either low-Pi (5 μM) or high-Pi (500 μM) media, and the rest of the root system in contact with non-[^14^C]IAA-containing low-Pi (5 μM) or high-Pi (500 μM) media. The ^14^C content of 2mm root sections starting 1mm from the root apex, divided by the ^14^C content of 2mm root sections starting 3mm from the root apex, expressed as a proportion of the high Pi controls. Values are the average of three biological replicates (four pooled roots from distinct organisms per replicate). Error bars are the standard errors (*n*=3). Letters indicate values significantly different from each other using Student’s *t*-test (*P*<0.05); two-way ANOVA determined that both tip phosphorus, basal phosphorus, and the interaction between the two had significant effects on the results (*P*<0.001). (B) Relative expression levels of *PIN* gene candidates in *T. aestivum* seedlings grown in either low-Pi (5 μM) or high-Pi (500 μM) media. Each sample was normalized for actin and tubulin expression as detailed in the Materials and methods, and plotted as a value relative to each gene’s high-Pi value. Values are the averages of three biological replicates (10 pooled root tips per replicate), with each of these being a pooled average of three experimental replicates. Error bars are the SEM (*n*=3). Asterisks indicate where the low-Pi value is significantly different from the high-Pi value within each gene using Student’s *t*-test (*P*<0.05).

Radiolabelled [^14^C]IAA was used to assess the root’s capacity to transport auxin basipetally from the root apex. The results in Fig. 4A show that there was a significant reduction in basipetal auxin flow when the root tip was in contact with low-Pi medium compared with that containing high-Pi, whatever the basal medium Pi content. Furthermore, two-way ANOVA shows that both root tip Pi supply and basal root Pi supply have significant impacts on this measure of basipetal auxin flow, with a significant interaction between the two factors (*P*<0.001).

In the database searches for PIN auxin transporter sequences, two new sequences, and the previously annotated *TaPIN1* and *TaPIN2*, which had predicted amino acid sequences that were highly similar to the AtPIN2 probe sequence used, were identified. Quantitative RT–PCR measurements made on these sequences also showed reduced expression of *PIN3* and *PIN4* in seedlings grown in low-Pi media (Fig. 4B; Supplementary Fig. S1 at *JXB* online). This down-regulation of PIN gene expression, coupled with the reduction in [^14^C]IAA flow, provides evidence that auxin transport capacity was significantly altered in *T. aestivum* roots in response to low-Pi environments.

## Discussion

### Auxin fluxes in the root tip are affected by Pi availability, potentially driving alterations in root branching

The results presented herein shed new light on how *T. aestivum* roots integrate phosphorus availability into the processes driving lateral root production. Auxin is well established as a key component in the control of lateral root production. The basal flow of auxin in the lateral root cap and epidermis, and its subsequent accumulation in pericycle founder cells, is thought to drive lateral root branching and elongation (Dubrovsky *et al*., 2001, 2008; De Smet *et al.*, 2006), with disruption of this process inhibiting lateral root production (Fig. 1E; Casimiro *et al.*, 2001).

The results in Fig. 4A demonstrate that when *T. aestivum* roots are in a low-Pi environment the basal auxin flow is greatly reduced, and this potentially causes the reduced lateral root density observed in Fig. 1A. Root tip contact with low-Pi environments has previously been shown to have the capacity to drive the remodelling of a plant’s root system architecture (Svistoonoff *et al.*, 2007). The alterations in the root expression profile of *AUX/IAA* genes caused by the level of Pi supply shown in the present study (Fig. 3) point to a remodelling of the auxin response profile within the root system. The experiments performed here demonstrate a modulation of basipetal PAT, and the transcriptional regulation of *TaPIN* genes, which could potentially produce this altered auxin response profile. There are several downstream steps where PAT could be modulated further, such as PIN endosomal cycling (Geldner *et al.*, 2001; Huang *et al.*, 2010) or MDR*/*PGP–PIN interaction (Blakeslee *et al.*, 2007). However, the results in Fig. 4A provide evidence that there is a net effect of environmental Pi level on PAT when perceived both at the root apex and in basal portions of the root. These experiments do not provide evidence of how this effect of *PIN* transcription is enacted. However, given that a measurable difference in auxin flow occurs in previously Pi-sufficient plants within 1h, the implication is that a signalling process produces this effect rather than a more long-term nutrient shortage response.

The Pi–PAT interaction demonstrated in the present study adds to the Pi–auxin interactions previously documented in other species. Auxin sensitivity modulation in response to Pi status has been previously demonstrated to occur in *Arabidopsis* roots by up-regulation of *TIR1* auxin receptor expression (Pérez Torres *et al.*, 2009). This is proposed to cause the increased lateral root and root hair density and reduced primary root growth characterized by the low-Pi response in *Arabidopsis* (Ma *et al.*, 2001). Given the importance of root hair production in phosphorus uptake (Bates and Lynch, 2001; Zhu *et al.*, 2010), it would be beneficial for the plant’s nutrition for the control of root hair plasticity to continue unabated under low-Pi conditions. In *T. aestivum* a reduction of basipetal PAT in low-Pi conditions is shown (Fig. 4A), yet previous experiments have demonstrated no effect of varying Pi conditions on root hair density (Ewens and Leigh, 1985). This could be explained by the spatial separation of the basal meristem where lateral root founder cells are specified and the differentiated tissues where root hairs are produced. However, the scarcity of information on Pi effects on *T. aestivum* root hair density, due to the large variability in root hair production between cultivars (Wu and He, 2011), hinders making definitive conclusions.

### *Triticum aestivum* Pi scavenging responses differ from those of the model plant *Arabidopsis*

These experiments also highlight the imprecision of extrapolating nutrient scavenging responses from the model plant *Arabidopsis* into other species. Previous studies using *Arabidopsis* and *H. vulgare* have shown that under low-Pi conditions the primary root meristem undergoes a process of terminal differentiation, whilst the maturation rate of LRPs is enhanced (Linkohr *et al.*, 2002; López-Bucio *et al.*, 2002; Sánchez-Calderón *et al.*, 2005; Huang *et al.*, 2008). Following this, PAT reduces after ~11 d, which could potentially be related to the terminal differentiation of the meristem and root cap, providing a reduction in the density of LRPs and the continued elongation of the remaining emerged lateral roots (Nacry *et al.*, 2005). Ten days after germination in the present study, growth of young *T. aestivum* seminal roots after germinationcontinued unabated (Fig. 2A, B, E), with significant limitation to maximum lateral root length (Fig. 2C, D, F) and lateral root density (Fig. 1), which is consistent with observations in long-term studies of other crop species (Borch *et al.*, 1999).

Pi is usually found in largest quantities in the topsoil, and therefore enhanced exploration of this area is beneficial to a plant subject to Pi deficiency (Zhu *et al.*, 2005). A short-term enhancement in lateral root production can be an effective method of increasing topsoil exploration, and this is reflected in the increased lateral root production and lateral root growth relative to that of primary roots in low-Pi conditions observed in some studies (Linkohr *et al.*, 2002; Huang *et al.*, 2008). However, in plants with fibrous root systems, such as *T. aestivum*, the production of a multitude of seminal and crown roots at varying angles from the seed/hypocotyl affords an alternative method of topsoil exploration. This has been demonstrated in *Solanum lycopersicum*, where low Pi conditions caused a significant increase in the number of adventitious roots in a process mediated by ethylene (Kim *et al.*, 2008). Unfortunately, within the timeline of this study, the number of seminal roots was still very low (3–4) and therefore did not show any significant alterations in number. Nevertheless their presence possibly de-emphasizes the importance of the explorative function of lateral roots, and means that their chief benefit is to modulate the root surface area in response to more local environmental stimuli. A switch to a root system dominated by lateral roots has been shown to enhance Pi uptake efficiency greatly (Zhu and Lynch, 2004); therefore, a deeper understanding of the molecular mechanisms behind the production of lateral roots is potentially of great importance for both targeted crop breeding and localizing application of fertilizers to improve uptake efficiency.

### *PIN* candidates

This study also presents new insights into the *PIN* gene family within *T. aestivum*, identifying candidates from published cDNA libraries and marking expression locations for two family members. PIN proteins are characterized by two hydrophobic domains, each containing five transmembrane helices, connected by a hydrophilic domain presumed to protrude into the cytoplasm (Křeček *et al.*, 2009). The predicted amino acid sequences of the genes used in this study, *TaPIN1*, *TaPIN2*, *TaPIN3*, and *TaPIN4*, contain the N-terminal hydrophilic domain and the hydrophobic domain, complete with five transmembrane helices, and *PIN1* and *PIN2* also contain the C-terminal hydrophobic domain (Supplementary Fig. S1 at *JXB* online). The absence of a C-terminal hydrophobic domain in *PIN3* and *PIN4* cDNA sequences was attributed to the incomplete nature of the sequences. All the amino acid sequences also each contain two di-acid motifs, involved in trafficking from the endoplasmic reticulum, and a tyrosine-based internalization motif, for recruitment into clathrin-dependent vesicles (Supplementary Fig. S1). Both of these features are characteristic of *PIN* genes in other species (Chawla and DeMason, 2004; Schnabel and Frugoli, 2004; Křeček *et al.*, 2009; Zhou *et al.*, 2011; Watanabe *et al.*, 2012), and so give more credence to the notion that these sequences encode *T. aestivum* PIN proteins. As only a limited portion of these cDNA sequences is available, it remains unclear as to which subgroup of PIN proteins TaPIN3 and TaPIN4 belong.

### *AUX/IAA* candidates

A study identifying various family members of the *AUX/IAA* gene family in *T. aestivum* has already been published (Singla *et al.*, 2006), and the *AUX/IAA* candidate cDNAs used in Fig. 3 add to this. The methodology used here identified the complete sequence of *IAA1* published by Singla *et al.* (2006) alongside the candidate sequences, but the other results identified in their study did not score as highly using the present methodology. This is perhaps due to the differences between using *Oryza sativa* AUX/IAA and *Arabidopsis* AUX/IAA amino acid sequences as the query sequence in the BLAST search. There are four conserved domains that are characteristic of AUX/IAA proteins identified in other organisms (Dharmasiri and Estelle, 2004; Jain *et al.*, 2006) and in *T. aestivum* (Singla *et al.*, 2006). The amino acid sequences predicted from the candidate cDNAs used in this study all contained domain III and IV, and a STOP codon at the C-terminus. These sequnces all either also contained domain I and II, or were incomplete sequences missing the N-terminal portion of the sequence (Supplementary Fig. S2). The modulation of *AUX/IAA* expression shown in Fig. 3 is the first example of a Pi-modulated auxin response shift that has been demonstrated in *T. aestivum*. The conclusion from these data when viewed in conjunction with the [^14^C]IAA transport data in Fig. 4A is that the alteration in PAT auxin flow causes a corresponding alteration in auxin responses, and therefore *AUX/IAA* expression levels. Figure 3 shows that *IAA3* expression appears to be positively correlated with Pi supply. As Fig. 4A shows that Pi supply significantly influences the basipetal flow of auxin, this may indicate that *IAA3* expression is localized to the basal regions of the RAM auxin maximum. However, as mapping the specific locations of *AUX/IAA* expression is not covered in this study, further work is required to verify this.

### Conclusions

**Fig. 5.**
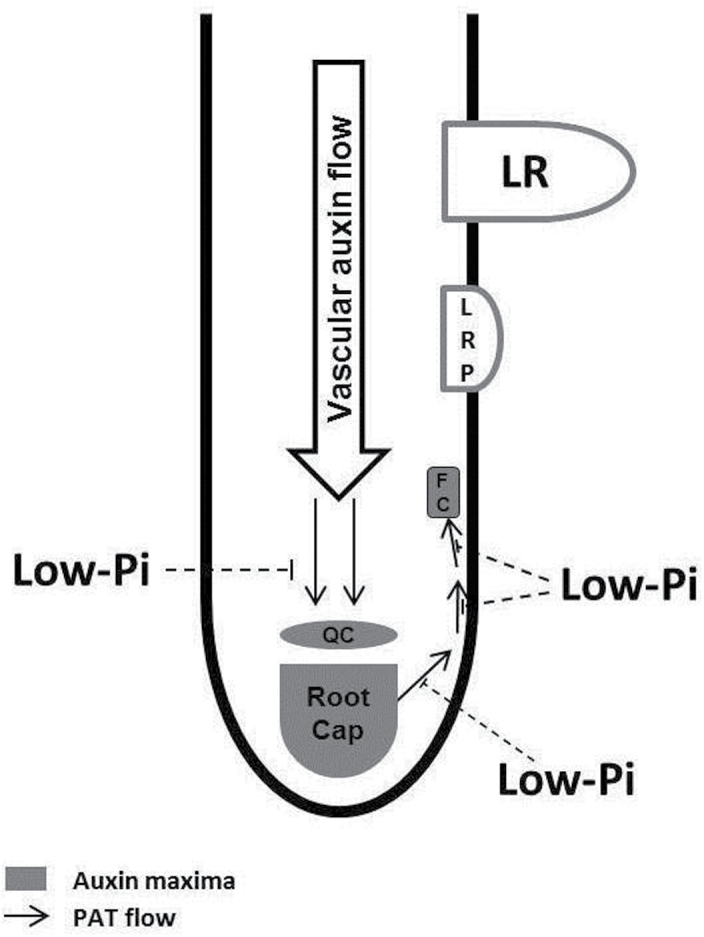
Environmental Pi modulates basipetal flows in polar auxin transport (PAT) to alter lateral root production. The inverted fountain of PAT (black arrows) creates auxin maxima (grey) at the quiescent centre (QC), root cap, and at founder cells (FC) in the distal meristem. LR, lateral roots; LRP, lateral root primordia. This study shows that low Pi acts through *PIN* expression to modulate the basipetal portion of this auxin flux.

The results presented here illustrate that the Pi-dependent modulation of auxin transport, driven by putative *PINOID* auxin export carrier gene expression, alters the auxin responses at the root tip. This is corroborated by a corresponding alteration in the root tip *AUX/IAA* expression profile, providing a potential mechanism for the decreased root branching observed in *T. aestivum* grown in low-Pi environments (Fig. 5). This significantly advances our understanding of the mechanism by which the developmental plasticity of the *T. aestivum* root system exploits heterogeneous soil environments. This is a potential mechanism for the widely observed phenomenon of localized branching in response to localized hotspots of soil phosporus (i.e. as would occur with banded Pi fertilization). Beyond advancing knowledge of plant biology, these findings have implications for the agricultural sector. Improved understanding of the mechanisms underpinning nutrient-stimulated root branching could improve targeting of agricultural fertilizers to regions where dense root branching is more probable, and highlights molecular mechanisms that could be exploited through plant breeding to improve existing varieties. There has also been a recent trend towards inoculation of agricultural plants with plant growth-promoting microorganisms, including auxin producers (Lugtenberg and Kamilova, 2009). Further understanding of the consequences of exogenous auxin application in crop species is therefore highly desirable. In conclusion, the present findings provide an understanding of the role of auxin in regulating root nutrient responses which should permit the more effective design of agricultural systems through combination of crop breeding and Pi fertilization regimes targeted at enhanced food security and the sustainable intensification of cropping systems.

## Supplementary data

Supplementary data are available at *JXB* online.

Figure S1. Sequence alignment of TaPIN3 and TaPIN4 amino acid sequences, displaying functional PIN protein motifs.

Figure S2. Alignment of the amino acid sequences of TaIAA candidates, displaying functional AUX/IAA domains.

Figure S3. AUX/IAA candidate sequence expression is elevated in response to 1h induction with auxin.

Table S1. Primers pairs used for qPCR analysis.

Supplementary Data
