# Supplementary material for: Phosphate depletion modulates auxin transport in Triticum aestivum leading to altered root branching
Source: J Exp Bot. 2014 Aug 2;65(17):5023–32. doi: 10.1093/jxb/eru284 (PMC4144783; doi:10.1093/jxb/eru284)
Supplement: Supplementary Data [file supp_65_17_5023__index.html]

Phosphate depletion modulates auxin transport in Triticum aestivum leading to altered root branching — Phosphate depletion modulates auxin transport in Triticum aestivum leading to altered root branching — Supplementary Data 

# Phosphate depletion modulates auxin transport in *Triticum aestivum* leading to altered root branching

## Supplementary Data

Data files

**Files in this Data Supplement:**

- Supplementary Data - Supplementary Data
