# Supplementary material for: Phosphate depletion modulates auxin transport in Triticum aestivum leading to altered root branching
Source: J Exp Bot. 2014 Aug 2;65(17):5023–32. doi: 10.1093/jxb/eru284 (PMC4144783; doi:10.1093/jxb/eru284)

Title: **Phosphate depletion modulates auxin transport in *Triticum aestivum* leading to altered root branching**

Authors: Peter J Talboys*, John R Healey, Paul J A Withers, Davey L Jones

*Corresponding author

Supplementary Table 1

Gene Candidate	Direction	Sequence 5' -> 3-	Accession #
ACTIN	F<br>R	ACCTTCAGTTGCCCAGCAAT<br>CAGAGTCGAGCACAATACCAGTTG	AB181991
TUBULIN	F<br>R	TGAGGACTGGTGCTTACCGC<br>GCACCATCAAACCTCAGGGA	U76558
IAA 1	F<br>R	TGACAACAGTAGAATAAAATCACCAAGTAG<br>GGTGGACCAGAAGCAATAATCTATC	AJ575098
IAA 2	F<br>R	CTGTTGCCAAGGCTCAGGT<br>TCACCAACCAGCATCCAGTC	CK213604
IAA 3	F<br>R	TACCGACAGCCTCCTCATGG<br>TCCTCGTAGGTGATGGCGTA	CK170519
IAA 4	F<br>R	ACCTCAGGAAGGTGGATCTC<br>TCTGGTTCTTACTCTTCTCGGC	CK163783
IAA 5	F<br>R	CTGAAGAGCAGCAAGGAGGAT<br>TTCTTGGACTTAACCCCTGCC	BI751049
IAA 6	F<br>R	TACTGCTAACGTCCCGCACA<br>TATCTTGGGGCCATTCCAGCA	AK332471
IAA 7	F<br>R	AACCAGGTGAAGAGCAGCAA<br>TTCTTATTCTTGGACTTGACCCCT	AK331670
IAA 8	F<br>R	AAGCCACACAAGATCACCTCG<br>GAGAAGCAGACGAACATGGC	AK330790
PIN 1	F<br>R	CCATCACGCTCTTCTCCCTC<br>CTCGATCATGTCCCTCCTGC	AY496058
PIN 2	F<br>R	CTGATGCAGGGCTAGGGATG<br>CGCCACGAGCATTCCAAAAA	BK005137
PIN 3	F<br>R	GATCACGGGCACGGACTTCT<br>GGCGCTCCTCCTCGTATTGG	CK208792
PIN 4	F<br>R	CATGATCCTCGCCTACGGCT<br>AACAATTGGAGTCCTCCCCCT	CK208849

Supplementary Figure 1

```
AY496058 (PIN1) > MITGTDFYHVMTAMVPLYVAMMLAYGSVKWWGIFTPDQCSGINRFVALFAVPLLSFHFIS
BK005137 (PIN2) > MITGKDIYDVLAAVVPLYVAMFMAYGSVRWWGIFTPDQCSGINRFVAVFAVPLLSFHFIS
CK208792 (PIN3) > MITGTDFYHVMTAVVPLYVAMILAYGSVKWWGIFTPDQCSGINRFVALFAVPLLSFHFIS
CK208849 (PIN4) > MIAGTDFYHVMTAVVPLYVAMILAYGSVKWWGIFTPDQCSGINRFVALFAVPLLSFHWIS   60

AY496058 (PIN1) > TNNPYTMNLRFIAADTLQKLIVLALLTLWSHLSRN------------GSLEWTITLFSLS
BK005137 (PIN2) > TNDPYAMDYRFLAADSLQKLVILAALAVWHNVLSRYRCRGGTEAGEASSLDWTITLFSLA
CK208792 (PIN3) > TNNPYTMNLRFIAADTLQKLMMLAMLTAWSHLSRR------------GSLEWTITLFSLS
CK208849 (PIN4) > GNNPYTMNLRFIAADTLQKLMMLAMLTGWSHLSRR------------GRLEWTITLFSLS   120

AY496058 (PIN1) > TLPNTLVMGIPLLKGMYGDESGSLMVQIVVLQCIIWYTLMLFMFEYRGARIPINRAVPDT
BK005137 (PIN2) > TLPNTLVMGIPLLRAMYGDESGSLMVQIVVLQSVIWYTLMLFLFEYRGAKALISEQFPPD
CK208792 (PIN3) > TLPNTLVMGIPLLKGMYGDESGSLMVQIVVLQCIIWYTLMLFMFEYRGARMLITEQFPDT
CK208849 (PIN4) > TLPNTLVMGIPLLKGMYGDESGRLMVQIVVLQCIIWYTLMLFMYEYRGARMLITEQFSDT   180

AY496058 (PIN1) > AGA-IASIAVDPDVMSLDGRRDMIETEAEVKEDGKIHVTVRRSN---------ASRSDIY
BK005137 (PIN2) > VGASIASFRVDSDVVSLNGR-EALHADAEVGRDGRVHVVIRRSASGSTTGGHGAGRSGIY
CK208792 (PIN3) > AGA-IASIVVDPDVVSLDGRSNAIETEAEVKEDGKIHVHVRRSS---------ASRSDIY
CK208849 (PIN4) > AGA-IASIVVDPEVVSLDGRSNAIETEAEVKDDGKIHVNVRRSS---------ASRSEIY   240

AY496058 (PIN1) > SRRSMGFSSTTPRPSNLTNAEIYSLQSSRNPTPRGSSFNHTDFYSMVGRSSNFAAGDAFG
BK005137 (PIN2) > RGAS---NAMTPRASNLTGVEIYSLQTSREPTPRQSSFNQSDFYSMFNGSK--LASPKGQ
CK208792 (PIN3) > SRRSMGFSQHTPRPSKLTNAEIYSLQSSRDPTPRGSTF----------------------
CK208849 (PIN4) > AGA-IASIVVDPEVVSLDGRSNAIETEAEVKDDGKIHVNVRRSS---------ASRSEIY   300

AY496058 (PIN1) > PVVRTGATPRPSNYEEDKAGN-NNNSKYGQYPAPNPAMAAPQKPAKKAANGQAKGEDGKD
BK005137 (PIN2) > PPVAGGGGARGQGLDEQVANKFKGGEAAAPYPAPNPGMMMPAP--RKKELGGSNSNSNKE
CK208792 (PIN3) > ------TTPTSTPWSA----------------------AAPTR--RRRRVRGRTGPRG--
CK208849 (PIN4) > ------GVADGTGFER-----------------------GPTR--ARSI-----------   360

AY496058 (PIN1) > LHMFVWSSSASPVSDVFGNG----------------------------------TEAYND
BK005137 (PIN2) > LHMFVWSSSASPVSEANLRNAVNHAASTDFAAAPPAAATPRDGATPRGVSGSVTPVMKKD
CK208792 (PIN3) > ------------------------------------------------------PVQYEE
CK208849 (PIN4) > ------------------------------------------------------------   420

AY496058 (PIN1) > AAAKDVRVAAASPRKADGVERDEFSFGNKERDAEAGDEKAAAEQGTAGLVAAPTVMPPTS
BK005137 (PIN2) > ASSGAVEVEIEDGMMKSPATGLGAKFPVSGSPYVAPRKKGADVP---GLEEAAHPMPPAS
CK208792 (PIN3) > -----------------------------ERPAQHPRPRGQNRPETAGHGEAP-------
CK208849 (PIN4) > -----------------------------MRRTPQPKPRGPKGPKRRRNGKARGRTP---   480

AY496058 (PIN1) > VMTRLILIMVWRKLIRNPNTYSSLIGLIWSLVCFRRNFVMPAIIMKSIAILSDAGLGMAM
BK005137 (PIN2) > VMTRLILIMVWRKLIRNPNTYSSLIGLVWSLVSFRWNIQMPTIIKGSISILSDAGLGMAM
CK208792 (PIN3) > ------------------------------------------------------------
CK208849 (PIN4) > ------------------------------------------------------------   540

AY496058 (PIN1) > FSLGLFMALQPRIIACGNKRATFAMAVRFLTGPAVMAAASIAVGLRGTLLQIAIVQAALP
BK005137 (PIN2) > FSLGLFMALQPKIISCGKSVATFAMAVRFLTGPAVIAATSIAVGLRGVLLHVAIVQAALP
CK208792 (PIN3) > ----------------------------------------LVVGLHG-----------VP
CK208849 (PIN4) > ----------------------------------------IVLWISG------ASRFEIF   600

AY496058 (PIN1) > QGIVPFVFAKEYSVHPDILSTAVIFGMLIALPITLVYYILLGL-----------------
BK005137 (PIN2) > QGIVPFVFAKEYNCHPQILSTAVIFGMLVALPITILYYVLLGI-----------------
CK208792 (PIN3) > GG--------SLGGSPT-------------------------------------------
CK208849 (PIN4) > GG----------------------------------------------------------   660
```

Supplementary Figure 2

```
AJ575098 (IAA1)> ------MAGADVDV------------------------GTELRLGLPGGGAEAAKAGKRG
CK213604 (IAA2)> -----MEATDSLLM------------------------ATELRLGLPG---------TDD
CK170519 (IAA3)> ----------------------------------------------------MRRTRSSP
CK163783 (IAA4)> ------------------------------------------------------------
BI751049 (IAA5)> ------------------------------------------------------------
AK332471 (IAA6)> MPPPNLEARDYIGLGPAAPAPSASSCSSSAS--GEAGPHLALRLGLPG---------RDE
AK331670 (IAA7)> MSPP-LEPHDYIGL--SAPTPSSSSCSSSPSPAAEAGPRLTLRLGLPGSESPDRDRDRDG
AK330790 (IAA8)> -----MEATDSLLM------------------------ATELRLGLPG---------TDD

AJ575098 (IAA1)> YEDTIDLKLTL---------PTGGMQEDSAGKPEPAADKAKRPAEAAAAD----------
CK213604 (IAA2)> KPHKITSVMSP---------PATPRGRKRTLDAFEATASDEADRSDDVET----------
CK170519 (IAA3)> PPPSLLPRLRW---------WDGHQSAAIARARMPTTTNQVKSSKEDSDT----------
CK163783 (IAA4)> -----------------------------------MATNLSAPRSKDEAE----------
BI751049 (IAA5)> ---------------------------------MATTTNQLKSSKEDXDT----------
AK332471 (IAA6)> PEAAVDAALTLGPAPATANVPHRGGAKRGFADSLLDRTAPAAGAGGEEDKQKGEAAAAAG
AK331670 (IAA7)> PADDVAPALTLGPA------PHKAASKRAFPDASPRRGCSAAARAEDKPP----------
AK330790 (IAA8)> KPHKITSVMSP---------PATPRGRKRTLDAFEATASDEADRSDDVET----------

AJ575098 (IAA1)> PEKPPAPKAQAVGWPPVRSYRRNAM--TVQSVKIKKEEETEKQQPAAAAAAGANGSNFVK
CK213604 (IAA2)> --APPVAKAQVVGWPPVRSYRKSCFQAAASKSKAKKADEASSNNTPSAAAPASTNGSFVK
CK170519 (IAA3)> ----------------------------------KQGQEF--------------- YVK
CK163783 (IAA4)> PKQAPAP-----------------------------------------------GCLYVK
BI751049 (IAA5)> ----------------------------------KXGQEF----------------LYVK
AK332471 (IAA6)> AGAPPAAKAQVVGWPPVRSYRKNTL--AANATKTKAENEG----------RSEAGCCYVK
AK331670 (IAA7)> SAAPPAAKAQVVGWPPVRNYRKNTL--AASASKAKAGDDG----------APH----YVK
AK330790 (IAA8)> --APPVAKAQVVGWPPVRSYRKSCFQAAASKSKAKKADEASSNNTPSAAAPASTNGSFVK

AJ575098 (IAA1)> VSMDGAPYLRKVDLKMYNTYKDLSIALQKMFSTFTA-----TGNEG-------KMVEAVN
CK213604 (IAA2)> VSMDGAPYLRKVDMRMYKGYRELREALEAMFVCF-------SGADG------GASNGGAN
CK170519 (IAA3)> VSMDGAPYLRKVDLKTYKNYKGMVVGLGKMFIGFR------TGKDG--------------
CK163783 (IAA4)> VSMDGAPYLRKVDLKMYKNYKDLSLELEKKFSGFTVGHGESTGKSGRDGLSDCRLMDLKS
BI751049 (IAA5)> VSMDGAPYLRKVDLKTYKNYKDMVVGLGKMFIGFR------TGKDG--------ASENRK
AK332471 (IAA6)> VSMDGAPYLRKVDLKTYSSYDNLSLELEKMFSCFITGKSSSCKTSTRDRLTDGSRADALQ
AK331670 (IAA7)> VSMDGAPYLRKVDLKMYSSYEDLSMALQKMFSCFITGQSSLRKPSTKDRLTNRSNVDSLQ
AK330790 (IAA8)> VSMDGAPYLRKVDMRMYKGYRELREALEAMFVCF-------SGADG------GASNGGAN

AJ575098 (IAA1)> GSDVVTTYEDKDGDWMLVGDVPWEMFVASCKRLRIMKGSEAIGLA-PRAKDKYKNKS
CK213604 (IAA2)> PAEYAITYEDKDGDLMLVGDVPFDMFSGTCKKLRIIKRSEATGLG-SK---------
CK170519 (IAA3)> --EYVMTYEDKDGDWMLVGDVPWEMFTESCRRIRVMKSSDVIGLGVTRAGVKSKNKN
CK163783 (IAA4)> GTELVLTYEDKDGDWMLVGDVPWRMFTDSCRRMRIMKGSDAVGLA-PRAAEKSKNQK
BI751049 (IAA5)> DGEYVMTYEDKDGDWMLVGDVPWEMFTESCRRIRVMKSADVXGLXVTRAGVKSKNKN
AK332471 (IAA6)> DQEYVLTYEDKDADWMLVGDLPWDLFTTTCRKLRIMRGSDAAGMA-PR---------
AK331670 (IAA7)> DQEYVLTYEDKDADWMLVGDLPWDLFTTICRKLKIMRGSDAAGIA-PRSLEQTGQNK
AK330790 (IAA8)> PAEYAITYEDKDGDLMLVGDVPFDMFSGTCKKLRIIKRSEATGLG-SK---------
```

Supplementary Figure 3

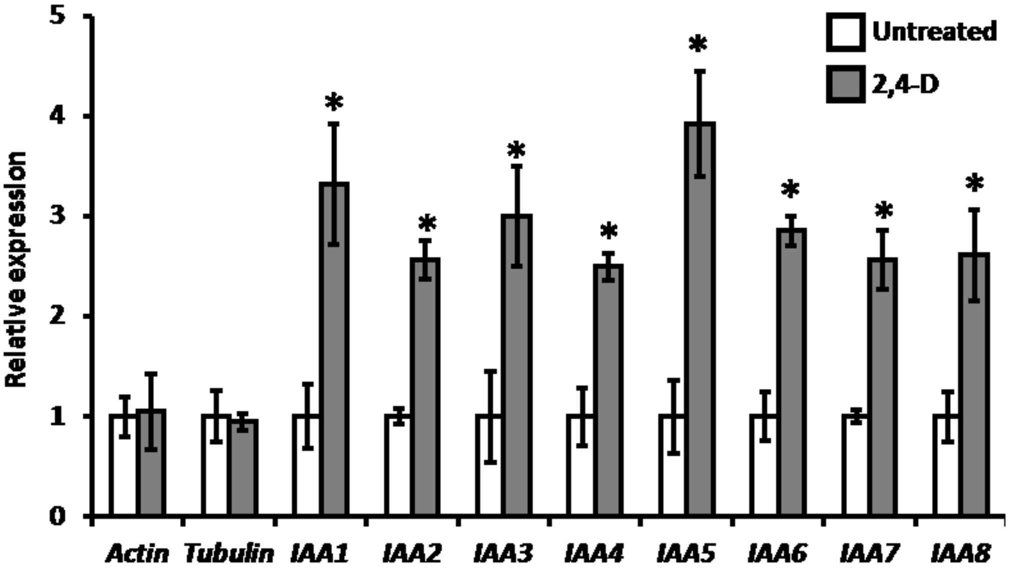

Supplement: Supplementary Data [file supp_eru284_jexbot124719_file001.pdf]
